# Critical Care Surge Capacity to Respond to the COVID-19 Pandemic in Italy: A Rapid and Affordable Solution in the Novara Hospital

**DOI:** 10.1017/S1049023X20000692

**Published:** 2020-05-19

**Authors:** Gianmaria Cammarota, Luca Ragazzoni, Fabio Capuzzi, Simone Pulvirenti, Nello De Vita, Erminio Santangelo, Federico Verdina, Francesca Grossi, Rosanna Vaschetto, Francesco Della Corte

**Affiliations:** 1. Anesthesia and General Intensive Care, “Maggiore della Carità” University Hospital, Novara, Italy; 2. Department of Translational Medicine, Università del Piemonte Orientale, Novara, Italy; 3. CRIMEDIM – Research Center in Emergency and Disaster Medicine, Università del Piemonte Orientale, Novara, Italy

**Keywords:** acute respiratory failure, COVID-19, crisis, surge capacity

## Abstract

The rapid insurgence and spread of coronavirus disease 2019 (COVID-19) exceeded the limit of the intensive care unit (ICU) contingency plan of the Maggiore della Carità University Hospital (Novara, Italy) generating a crisis management condition. This brief report describes how a prompt response to the sudden request of invasive mechanical ventilation (IMV) was provided by addressing the key elements of health care system surge capacity from contingency to crisis. In a short time and at a relatively low cost, a structural modification of a hospital aisle allowed to convert the general ICU into a COVID-19 unit, increasing the number of COVID-19 critical care beds by 107%.

## Background

The coronavirus disease 2019 (COVID-19) pandemic is now menacing several health systems across the globe. Currently, Italy has been one of the most affected countries where hospitals are still struggling to deal with the surge of patients.^1^ Although the majority of patients experiences mild or poor symptoms, in 14% of cases, a severe acute respiratory failure (ARF) may occur leading to intensive care unit (ICU) admission.^2^ Therefore, urgent actions are required to modify ICU’s capacity to deal with a massive influx of severe ARF patients who require intubation. In this regard, the rapid expansion of operational staff, stuff, and structures is paramount for health care system surge capacity.^3^ Moreover, it has been described that the categorization and implementation of different phases of surge capacity from conventional to contingency to crisis.^4,5^

This brief report describes the strategy implemented in the Maggiore della Carità University Hospital (Novara, Italy), the second largest third-level referral hospital of the Piedmont Region, to provide a prompt response to the steep growing demand for invasive mechanical ventilation (IMV) in course of COVID-19 pandemic.^6,7^

## Structure

**Figure 1.**
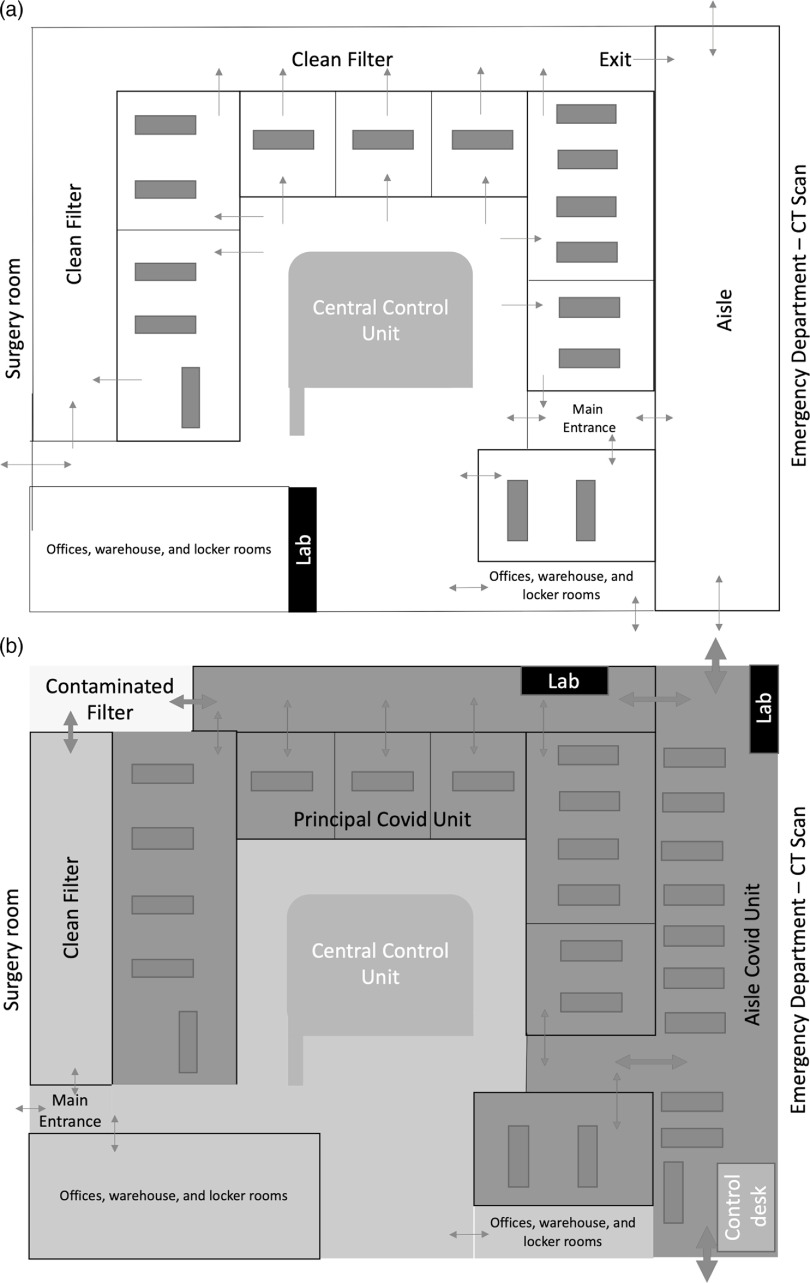
Intensive Care Unit Converted to COVID-19 Unit.

Figure 1a depicts the general ICU set-up during normal routine: 14 ICU stations and a shock room with two dedicated positions for in- and out-of-hospital emergencies are all placed around a central unit for visual control and telemetry monitoring. While COVID-19 was rapidly spreading in the Northern Italy, the ICUs were progressively converted into a dedicated, cohorted unit for COVID‐19‐positive patients requiring IMV. In parallel, all non-COVID-19 ICU patients were gradually transferred to an eight-bay post-anesthesia care unit, which was adapted into a general ICU. The activation of the hospital contingency plan for massive influx of patients established the interruption of deferrable surgical interventions and the adaptation of five operating rooms into 10 COVID-19 ICU stations distributed in different hospital locations. All urgent procedures and non-deferrable oncological interventions were securely maintained. However, during the days, the number of patients with severe ARF exceeded the limit of the contingency plan.

To meet the unexpected needs, a crisis plan was designed and implemented under the supervision of building engineers. As shown in Figure 1b, an aisle was transformed into an additional critical care area and connected to the principal ICU COVID-19 due to its proximity to the emergency department and the computer tomography scanner. Lasting only four days of work at a cost of €51,000 ($55,000), the project consisted in a structural modification of the aisle (ie, the realization of medical gases and vacuum supplying circuits, the ad hoc wiring configuration, the improvement of air recirculation system, and the installation of two main doors on both sides of the aisle for isolation). Locked from inside, these doors allow patients admittance and discharge, and trash disposal. Considering also the additional use of the shock room, this new configuration allowed the rapid creation of 12 additional critical care stations enlarging the principal COVID-19 ICU to 26 beds with a small sacrifice in ergonomic workspace. Three beds were preserved in one operating room for stabilizing the suspected cases before the admission to the principal ICU, where patients can only enter with a confirmed laboratory diagnosis of COVID-19. Wearing personal protective equipment (PPE), ICU staff can safely access the so-called “red zone” through a dedicated unidirectional entry/exit pathway (Figure 1b).

## Stuff

Within the aisle, all the new ICU stations were equipped with standard intensive care equipment (ie, bed, monitor, ventilator, suctioning system, and syringe driver with the appropriate stand). Moreover, additional critical care tools (ie, emergency cart, defibrillator, video-laryngoscope, disposable fiberscope, emergency cricothyroidotomy kit, chest drain set, ultrasound machine, refrigerator, and transport ventilator) were rapidly collected and stored in two dedicated areas (the so-called “lab”) to allow their rapid accessibility from different zones of the ICU (Figure 1b). The COVID-19 ICU was also equipped with an arterial blood gas analyzer, a machine for thromboelastographic assessment, a scialytic lamp, and an x-ray machine.

The communication between the red zone and the control unit is allowed by telephones installed ad-hoc in the red zone. The installation of ceiling video cameras in the aisle allows the supervision of the clinical activities from the control unit. Also, the ICU staff can add clinical notes and make changes in patient therapies through a dedicated software installed on tablets connected to the control unit without exiting the red zone and removing the PPE.

## Staff

The strategy also included an increase of ICU staff. While the general ICU were progressively converted to COVID-19 unit and the contingency plan was implemented, anesthesiologists, nurses, and other health care professionals, recruited from surgical teams, underwent a specific just-in-time training to improve technical skills in the application of PPE and in the clinical management of mechanically ventilated ARF patients. Furthermore, the staff was also trained to operate through a dedicated electronic medical records software. Once the training had been completed, the new personnel were flanked with highly experienced ICU staff ensuring a 10-day apprenticeship process. The staffing level was set with a 1:2 nurse-to-patient ratio and a 1:4 physician-to-patient ratio. However, being that the operating rooms converted into COVID-19 ICU stations, which were dispersed in different locations distant from the supervision of the control unit, the ICU staff had significant difficulties to work as one team and to ensure the same level of care. The structural modification of the aisle eliminated these important limitations homogenizing staff competencies and patient care.

## Conclusions

The rapid insurgence and spread of COVID-19 exceeded the limit of the ICU contingency plan for massive influx of patients generating a crisis management condition. By addressing the key elements of health care system surge capacity from contingency to crisis, a prompt response to the sudden request of IMV was provided, converting the general ICU into a COVID-19 unit and increasing the number of COVID-19 ICU beds by 107%. In a short time and at a relatively low cost, the structural modification of the aisle allowed to simplify the supervision of the clinical activities and increased the level of quality of care with only a small reduction in ergonomic workspace.
